# Responses to Raven matrices: Governed by visual complexity and centrality

**DOI:** 10.1177/03010066231178149

**Published:** 2023-06-02

**Authors:** Joost C. F. de Winter, Dimitra Dodou, Yke Bauke Eisma

**Affiliations:** 2860 Delft University of Technology, The Netherlands

**Keywords:** Raven Advanced Progressive Matrices, eye tracking, perception, visual complexity, attention distribution

## Abstract

Raven matrices are widely considered a pure test of cognitive abilities. Previous research has examined the extent to which cognitive strategies are predictive of the number of correct responses to Raven items. This study examined whether response times can be explained directly from the centrality and visual complexity of the matrix cells (edge density and perceived complexity). A total of 159 participants completed a 12-item version of the Raven Advanced Progressive Matrices. In addition to item number (an index of item difficulty), the findings demonstrated a positive correlation between the visual complexity of Raven items and both the mean response time and the number of fixations on the matrix (a strong correlate of response time). Moreover, more centrally placed cells as well as more complex cells received more fixations. It is concluded that response times on Raven matrices are impacted by low-level stimulus attributes, namely, visual complexity and eccentricity.

Raven matrices are a test of abstract reasoning completed by hundreds of thousands of participants in published research over the past few decades (Brouwers et al., 2009). They were designed to measure the ability to think abstractly and solve problems without relying on prior knowledge or experience, in an attempt to measure “true intelligence” (Penrose & Raven, 1936). The Raven matrices have also been argued to be culture-free (Jensen, 1998; Valencia, 1979), meaning that they do not rely on specific knowledge or skills that may be more common in one culture than another. Finally, Raven matrices were designed to be independent of language, so that individuals from different language backgrounds can take the test without any disadvantage. Research has shown that performance on Raven matrices correlates strongly with the general intelligence factor (*g*) (Marshalek et al., 1983; Tucker-Drob & Salthouse, 2009). According to Gignac (2015), on the other hand, Raven matrices do not have special qualities that make them a test of true intelligence but are one of several good-quality tests that provide an indication of general intelligence.

Responses to Raven matrices are typically explained in terms of top-down analytical reasoning. For example, based on think-aloud and eye-tracking data, Carpenter et al. (1990) concluded that performance on Raven matrices reflects the ability of participants to induce abstract relations and problem-solving goals in working memory. A study using eye-tracking by Vigneau et al. (2006) found that the “proportion time eyes on matrix” was positively correlated with the number of correct responses and negatively correlated with response time, i.e., participants who focused more on the problem region tended to perform better and faster. This finding is consistent with the hypothesis that constructive matching (i.e., first constructing an idealized answer and then selecting that answer among the response alternatives) is an effective strategy for arriving at the correct response on Raven matrices. Rivollier et al. (2021) found similar effects by using a method that did not use eye-tracking but visual exploration using the computer mouse instead. Li et al. (2022) showed that, among test-takers who reported a constructive matching strategy, correlations with working memory capacity (measured through memory span tasks) were stronger than among those who reported they were using a response elimination strategy (i.e., examining the different response options to decide which one could be the missing piece). Gonthier and Roulin (2020) and Jarosz et al. (2019) found that the constructive matching strategy was especially used by participants with high working memory capacity measured through memory span tasks. Similarly, eye-tracking research by Loesche et al. (2015) showed that providing participants with specific rules about how to solve matrices decreased the number of saccades to the response alternatives, suggesting increased use of a constructive matching strategy.

At the same time, it is known that human attention is not only governed by top-down factors, such as strategies and goals, that are maintained in working memory but also by “bottom-up” visual factors. More specifically, salience, which can be defined in terms of image luminance contrast (e.g., edges), color contrast, changes in orientation, or texture, is an attention attractor (Itti et al., 1998; Nothdurft, 2000; Treue, 2003). Research on viewing paintings has shown that observers are more likely to look at elements that contain details as compared to more homogeneous surface areas (De Winter et al., 2022; DiPaola et al., 2010).

Visual demands may also play a key role in solving Raven matrices. In fact, the easier items of the Raven matrices (Standard Progressive Matrices) have been constructed to test basic perceptual processes, such as differentiation, recognition of similarity, and Gestalt perception (Raven et al., 1998). Additional support for the role of perceptual processing in solving the Raven matrices is offered by a number of authors. For instance, using manually constructed rules, DeShon et al. (1995) classified the items of Raven Advanced Progressive Matrices (RAPM) as visual (requiring superposition, movement, addition/subtraction, or rotation) or analytical (constant in a row, pairwise comparison, distribution of two or three values). Meo et al. (2007) let participants solve different versions of the RAPM: the original version versus modified versions that were identical in terms of the solution rules to be applied but visually more cluttered, where cells of the Raven matrices were replaced by invented or overlapping letters. Their results showed that, for easy Raven items in particular, participants provided their responses faster for the original Raven matrices than for items consisting of letters. Based on their findings, the authors concluded that perceptual processing plays a significant role in solving Raven matrices. Hua and Kunda (2020) and Kunda (2020) applied a so-called inpainting computer vision method on Colored Progressive Matrices. In inpainting, a missing part of an image is estimated using a pre-trained convolutional neural network. The authors found that a large proportion of the Raven matrices could be answered correctly using this inpainting approach, which suggests that solving Raven matrices, at least in part, involves low-level visual processing without requiring analytical reasoning. It can be argued that the strong focus on vision contradicts the design principles of the Raven matrices, which aim to measure fluid intelligence and abstract reasoning.

According to the salience, effort, expectancy, value (SEEV) model of Wickens and McCarley (2008), effort is another factor influencing the likelihood that a viewer glances at an area of interest. Based on this model, effort can be understood as the physical expense involved in redirecting attention, measured by the amount of eye and head movement needed to focus on a specific area of interest. It is suggested that individuals tend to favor shorter scans over longer ones, especially when the expected value of the shorter scans is similar (Wickens & McCarley, 2008). Research concurs that in distributed attention tasks, humans are more likely to glance at the center of the task environment, while being less inclined to glance at more eccentric regions, presumably because it is less effortful to do so (Eisma et al., 2018). The so-called central fixation bias also holds for other types of viewing tasks, such as viewing paintings or scenes on a computer monitor (Bindemann, 2010; Schütz et al., 2011; Tatler, 2007). In the case of Raven matrices, a scanning strategy where the center cell of the 3  ×  3 matrix is frequently visited and compared to surrounding cells can be expected to be less effortful than visiting edge cells often or sampling all cells with equal likelihood.

The aim of the current study is to examine how well participants’ responses can be predicted from the bottom-up factors “centrality” and “visual complexity.” Based on earlier works, like those by Meo et al. (2007) and Hua and Kunda (2020), we expected that Raven matrices, and individual cells of those matrices, that are visually more complex would attract more fixations and take a longer time to complete.

## Methods

### Participants

The Raven matrices were the last part of an experiment in which participants first looked at images of automated cars for about 8 min (Eisma et al., 2021) and subsequently performed a visual inspection-time task for about 5 min (Eisma & De Winter, 2020). Participants were 165 MSc engineering students. Six participants were removed because of missing or low-quality eye-tracking data (the same six participants were excluded in Eisma & De Winter, 2020). The remaining 159 participants were 50 females and 109 males, with a mean age of 23.52 years (*SD*  =  1.98). Thirty-three participants used visual aids during the experiment (23 contact lenses, 10 glasses). The research was approved by the TU Delft Human Research Ethics Committee, and all participants provided their written informed consent.

### Apparatus and Software

Movements of the left and right eyes were recorded at 2000 Hz using the SR Research EyeLink 1000 Plus. The experiment was programmed in the SR Research Experiment Builder.

Participants positioned their heads on a head support on the edge of a table. The monitor was positioned 95 cm from the edge of the table and at a horizontal distance of about 91 cm from the participants’ eyes. The eye-tracking camera was positioned 60 cm from the table's edge, with a horizontal distance of 56 cm between the camera lens and the participants’ eyes.

The stimuli were shown on a 24.5-inch BENQ monitor (XL2540) with a resolution of 1920  ×  1080 pixels and a refresh rate of 144 Hz. The monitor subtended horizontal and vertical viewing angles of approximately 33° and 19°, respectively. Participants wore closed-back headphones to block out ambient noise. The illuminance of the fluorescent lighting in the room, measured with a Konica Minolta T-10MA, was 390–410 lx, and the luminance of the monitor, measured with a Konica Minolta LS-150, was 70–75 cd/m^2^. The sensors were positioned near the head support and pointed toward the screen.

### Procedures

At the beginning of the experiment, participants completed EyeLink's standard nine-point calibration procedure. Before the start of the Raven matrices, participants were instructed on the computer screen as follows: “You will be asked to solve challenging visual-spatial problems. Your task is to solve as many problems as possible in 7 min. Each problem is a pattern with a bit cut out of it. You have to find the piece (numbered 1 to 8) that is needed to complete the pattern. The problems will get increasingly difficult.”

### Raven Matrices

**Figure 1.**
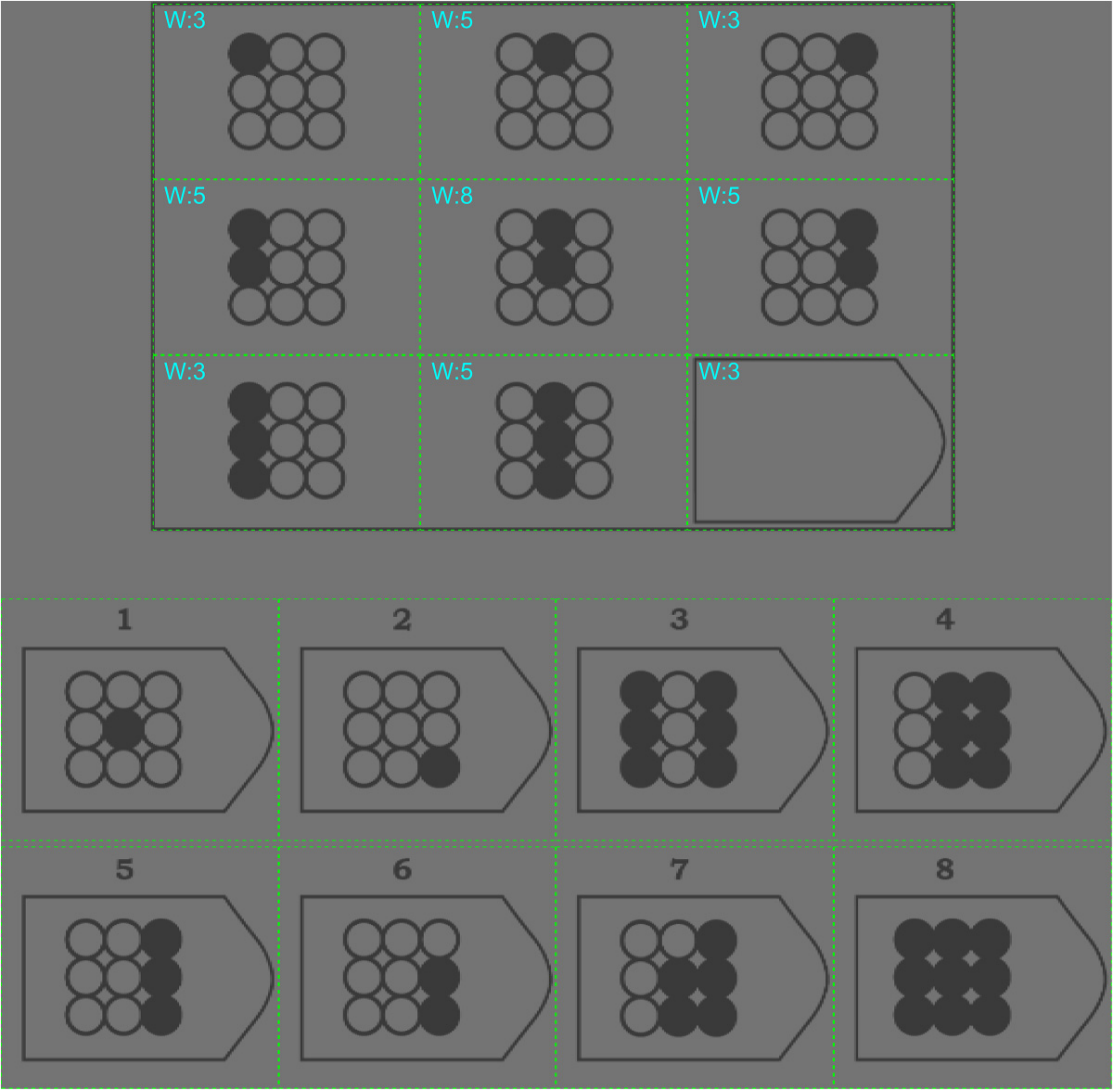
Example item of Raven matrices. The areas of interest (AOIs) are depicted as green outlines (not visible during the experiment). This item has been generated by the current authors to ensure that the original Raven matrices remain confidential and test integrity is preserved. Also shown are the centrality weights of the matrix cells used in regression analysis (described later in the Methods section).

Figure 1 shows an example of Raven item similar to those presented to the participants. The background was gray (RGB 127, 127, 127), and the shapes were darker gray (RGB 70, 70, 70). The low contrast presentation of the Raven matrices was used to limit the influence of extraneous factors, such as luminance changes, on the participants’ pupil diameter, blinking, and eye movements.

Participants were first given a practice item (RAPM Set 1, Item 7), for which they had 1 min. Participants were then provided with a 12-item version of Raven matrices (Arthur & Day, 1994; Arthur et al., 1999: RAPM Set 2, Items 1, 4, 8, 11, 15, 18, 21, 23, 25, 30, 31, and 35). Participants had 7 min to provide as many correct responses as possible. Participants could select a response by typing 1 to 8 on the keyboard, causing the response to be outlined. Upon pressing ENTER, they confirmed their response and immediately proceeded to the next Raven item.

### Data Processing

The horizontal (*x*) and vertical (*y*) gaze coordinates were averaged between the left and right eyes if both were available. Periods during which vertical gaze data on the screen were unavailable, as well as eye blinks, were labeled as data gaps. A 100-ms margin was added before and after each data gap, corresponding to the closing time and reopening time of the eyelid (Caffier et al., 2003). The data gaps in the gaze *x* and *y* coordinates were linearly interpolated. The *x* and *y* gaze coordinates were subsequently median-filtered using a window length of 100 ms.

The data were subsequently partitioned into fixations and saccades. A custom fixation filter was created that was inspired by Nyström and Holmqvist (2010). Gaze speed was computed in degrees per second and then filtered using a second-order Savitzky-Golay filter with a frame length of 31 samples (at 2000 Hz). Saccades were defined as occurrences where the gaze speed exceeded 30 deg/s. A minimum and maximum saccade duration of 10 ms and 150 ms was adopted, while the minimum fixation duration was 40 ms.

Eye-tracking data for unanswered Raven items were not taken into consideration. The following measures were used per Raven item per participant:

• *Response time (s).* The time it took the participant to enter their response.
• *Number of fixations per cell.* Fixations were counted on the AOIs surrounding the nine matrix cells and eight response alternatives (see Figure 1). If the fixation interval overlapped with a data gap, then the fixation was not included in the analysis.

**Figure 2.**
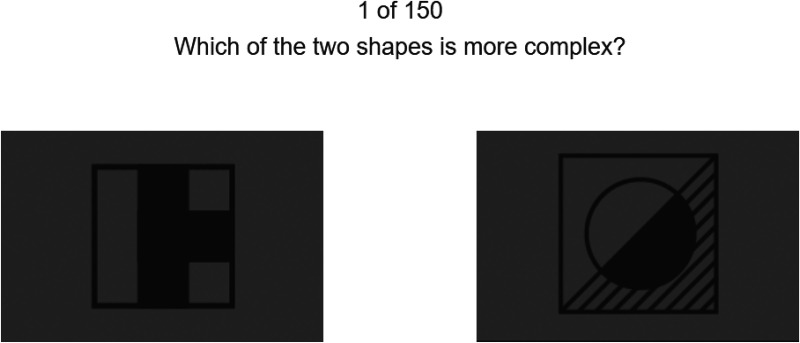
One of the pairwise comparisons completed by participants through crowdsourcing.

Furthermore, we examined whether the number of fixations directed to the matrix cells was associated with centrality (a measure of “effort”) and the visual complexity of that cell. Visual complexity was determined in two different ways:

1. *Edge density (0 to 1).* This measure represents the proportion of pixels that are edges and has previously been used as an index of visual clutter (Rosenholtz et al., 2007). It has also been found to correlate strongly with self-reported visual complexity (Forsythe et al., 2003; Machado et al., 2015). Edges were detected using Sobel's method (Sobel & Feldman, 1968), which detects edges at those points where the gradient of the image is maximum.
2. *Perceived complexity.* Automated measures of complexity, such as edge density, are not veridical representations of complexity as perceived by humans (Donderi, 2006; Nagle & Lavie, 2020). For example, a square filled with many parallel diagonal lines will have a high edge density but may be seen as relatively simple by humans because of its uniformity. On the other hand, humans may believe that shapes are complex if the shape consists of independent angles or curves (Attneave, 1957). We calculated a perceived complexity score for each cell based on a paired-comparison task implemented in Qualtrics. The task was completed by 599 participants recruited through the crowdsourcing platform Prolific. Each participant performed 150 randomly selected paired comparisons from the 96 matrix cells of the Raven items (12 Raven items  ×  8 matrix cells) (see Figure 2 for an example). A continuous complexity score was computed from the results of the pairwise comparisons using computer code provided by Pérez-Ortiz and Mantiuk (2017), without the use of distance priors. The complexity scores of the 96 cells have a mean of 0 and are scaled in such a way that a difference of 1 between two cells indicates that an approximated 75% of participants perceived the cell to be more complex than the other cell. Details about the crowdsourcing method are provided in the Supplementary Material.

The association between the number of fixations and both visual complexity and cell centrality was evaluated in three complementary ways:

1. *Between-item prediction.* It was examined whether the number of fixations directed to the matrix cells was predictable from the matrix cell complexity (i.e., edge density and perceived complexity) averaged across the nine cells of that item. Correlation coefficients were also computed between Raven item complexity and responses (mean response time, percentage of correct responses, and item number). Note that the Raven matrices are presented in a progressive order of difficulty, meaning that higher item numbers have previously been established among large samples to yield a smaller percentage of correct responses (e.g., Arthur & Day, 1994; Raven et al., 1998).
2. *Within-item prediction.* Second, by conducting a linear regression analysis, it was examined whether the number of fixations directed to the nine matrix cells could be predicted from the cells’ centrality and complexity. Cell centrality was defined using numeric weights, where the four edge cells (top left, top right, bottom left, bottom right) received a weight of 3, the middle edge cells (top middle, middle left, middle right, bottom middle) received a weight of 5, and the central cell received a weight of 8 (see Figure 1). The values 3, 5, and 8 represent the number of adjacent cells in the matrix. The reasoning behind these weights is that if a participant randomly samples adjacent cells of the matrix, the middle cell will be sampled most often (because it connects to eight neighboring cells), and the edge cells will be reached least often (because they connect to only three neighboring cells).

## Results

Of the 12 Raven items, participants on average had 7.30 correct responses (*SD*  =  1.93, min  =  2, max  =  11), made 3.48 mistakes (*SD*  =  2.35, min  =  0, max  =  10), and left 1.22 items unanswered (*SD*  =  1.43, min  =  0, max  =  6). The average time taken to enter the responses was 5.87 min (*SD*  =  0.90). This is shorter than the allotted 7 min because only 73 participants completed all 12 items in time, while the experiment was automatically terminated for the other 86 participants at 7 min (the unanswered items were not taken into consideration). Descriptive statistics per item are provided in the Supplementary Material.

### Distribution of Eye Gaze

**Figure 3.**
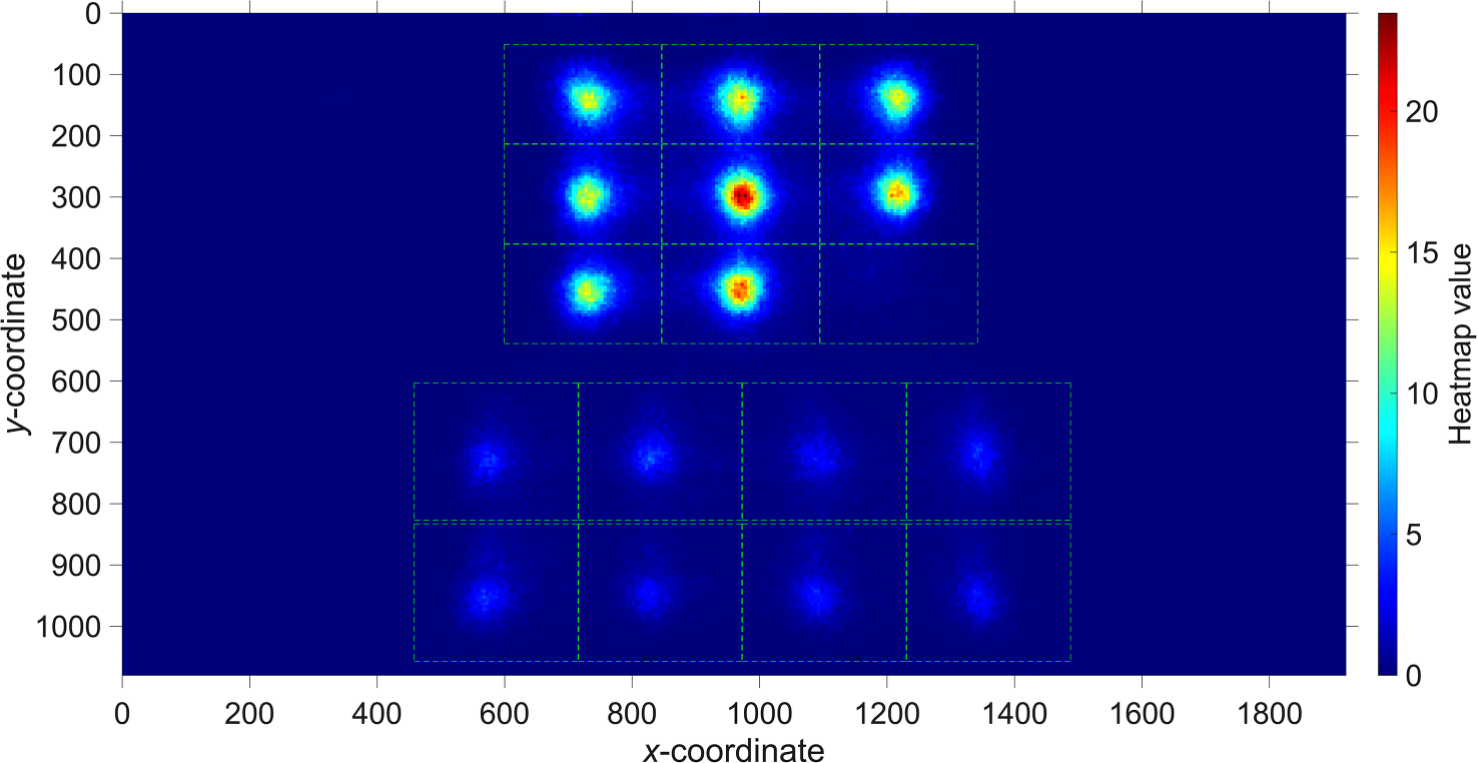
Heatmap of all collected eye-gaze data. The heatmap was created by dividing the screen into squares of 5  ×  5 pixels, and counting the number of samples (after downsampling to 100 Hz), the squares were gazed at. All counts were then divided by the number of participants (159).

Figure 3 shows a heatmap of the participants’ gaze points. It can be seen that the matrix cells received more attention than the response alternatives. Furthermore, the center matrix cell received more attention than the eight surrounding cells. The empty bottom right matrix cell received almost no attention.

### Edge Density and Perceived Complexity of the Matrix Cell

**Figure 4.**
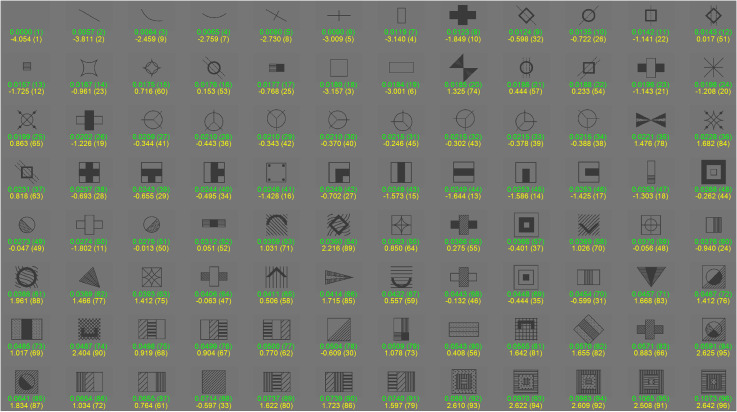
Matrix cells (96 in total, i.e., 12 Raven items  ×  8 cells per Raven item) sorted by edge density. Below each image, the edge density (in green) and perceived complexity (in yellow) are shown. Visual complexity ranks are shown in parenthesis, where 1 refers to the lowest value and 96 refers to the highest value.

Figure 4 shows the edge density of the matrix cells sorted in an ascending order. It can be seen that there were large differences in edge density; generally, shapes consisting of single lines, squares, rectangles, or small shapes had a low edge density, while large figures comprising many lines had a high edge density.

It is important to emphasize that the upper left cell of test item 9 was vacant, with an edge density of 0.000 and a perceived complexity score of −4.054. Additionally, the lower right matrix cell for all 12 Raven items was similarly empty, which is why it is not depicted in Figure 4. Consequently, the edge density and perceived complexity values for these lower right cells were set to 0.000 and −4.054, respectively.

Figure 4 also shows the results for the perceived complexity, calculated from the pairwise comparisons. The two measures of complexity were strongly associated (*r*  =  0.72, *n*  =  96), though discrepancies existed. For example, matrix cells consisting of many lines, such as the shape having an edge density of 0.0714 (ranked 88th of 96), were rated as relatively non-complex (corresponding perceived complexity  =  –0.597, ranked 33rd of 96). On the other hand, a cell consisting of a small number of curved lines, such as the image having an edge density of 0.228 (ranked 36th of 96), received a high perceived complexity score (perceived complexity  =  1.682, ranked 84th of 96).

### Between-Item Prediction of the Number of Fixations, Item Difficulty, and Mean Response Time

We calculated the edge density and perceived complexity scores for each of the 12 Raven items by taking the average of the values across the nine cells of the matrix. The correlation coefficients shown in Table 1 indicate that edge density and perceived complexity are statistically significant predictors of the mean response time.

**Table 1.** Correlation coefficients between complexity measures and difficulty measures and item response time measures among Raven items (*n*  =  12).

	Number of fixations on matrix area	Percentage of correct responses (%)	Raven item number (coded as 1 to 12)	Mean response time (s)
Edge density	*r* = 0.80, *p* = 0.002	*r* = –0.67, *p* = 0.016	*r* = 0.51, *p* = 0.094	*r* = 0.83, *p* < 0.001
Perceived complexity	*r* = 0.52, *p* = 0.082	*r* = –0.60, *p* = 0.041	*r* = 0.38, *p* = 0.221	*r* = 0.60, *p* = 0.038

**Figure 5.**
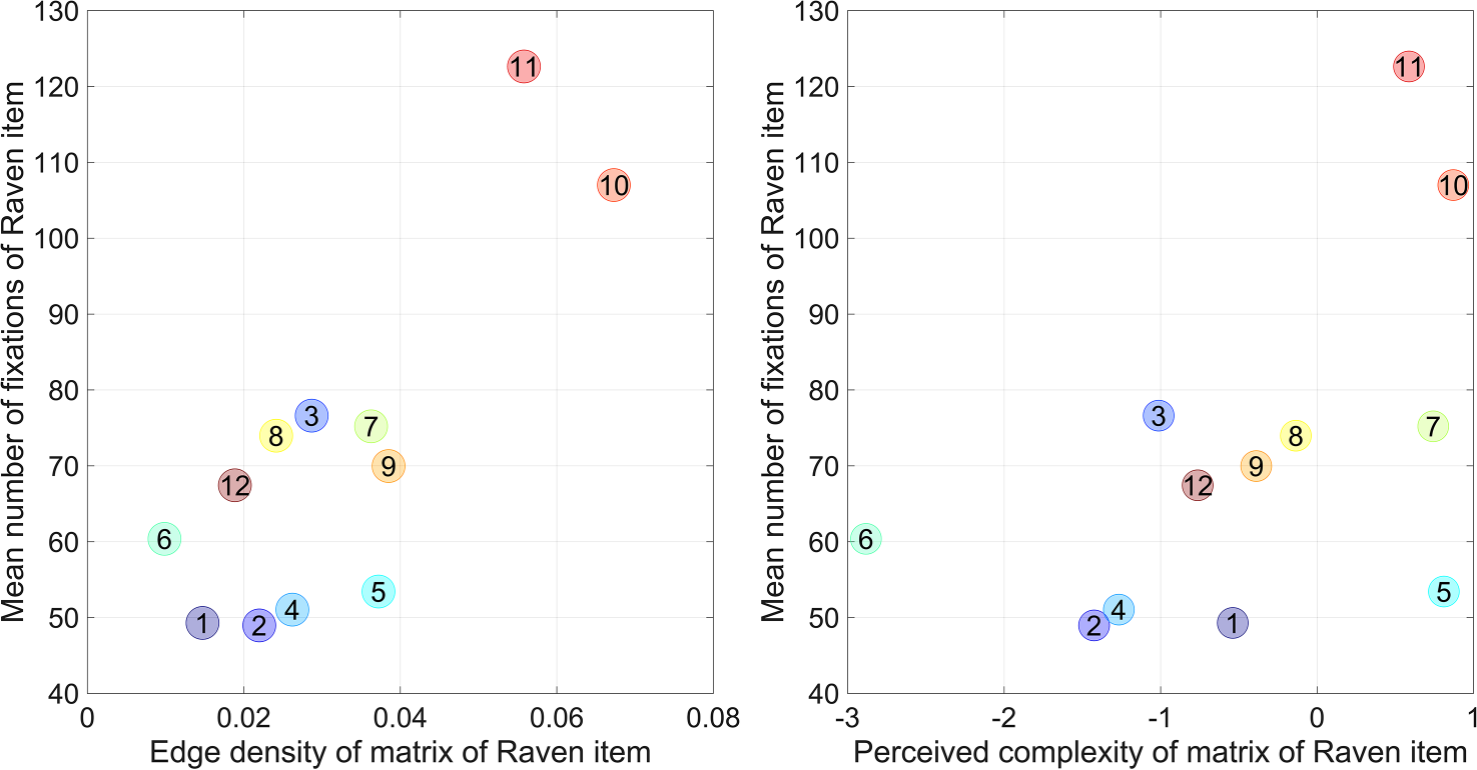
Mean number of fixations on matrix cells versus edge density per Raven item (left) and versus perceived complexity per Raven item (right). Edge density and complexity were computed from the mean of the nine matrix cells for that Raven item. The numbers in the circular markers indicate the test item number.

Moreover, edge density and perceived complexity were moderately predictive of Raven item difficulty, as measured by the percentage of correct responses and item number. These findings indicate that visually more complex Raven items were more difficult to solve and required longer response times. Figure 5 illustrates the correlation coefficients between complexity measures and the mean number of fixations on the matrix cells using scatter plots.

We also computed the partial correlation coefficient between edge density and mean response time while controlling for Raven item number (coded as 1 to 12). The partial correlation was found to be 0.82 (*p*  =  0.002). Similarly, the partial correlation coefficient between perceived complexity and mean response time, while controlling for Raven item number, was 0.53 (*p*  =  0.090). In other words, the predictive power of Raven item complexity remained similar to the correlations shown in Table 1, when item difficulty was controlled for.

The current study used a short 12-item version of the RAPM. However, the edge density can also be computed for the remaining items and then correlated with the response times observed for those items in the literature (for studies that provide response times per RAPM item, see Adam & Vogel, 2018; Ellis et al., 2021; Frischkorn & Von Bastian, 2021; Goldhammer et al., 2015; Gonthier & Roulin, 2020; Liu et al., 2022; Loesche et al., 2015; Poulton et al., 2022; Rivollier et al., 2021; Robison & Campbell, 2023; Sense et al., 2019; Tsukahara & Engle, 2021, 2023; and Vigneau et al., 2006). If using this approach, the edge density correlated between 0.27 and 0.73 with the mean response time, and Raven item number (1 to 36) correlated between 0.51 and 0.87 with the mean response time (see Supplementary Material). The partial correlations between edge density and mean response time, while controlling for Raven item number, were positive in 14 of the 15 studies, again indicating that edge density had predictive value beyond item difficulty (item number). These partial correlations were between −0.02 and 0.70, and although positive in the majority of studies, they were smaller than in our study (0.80). This could be explained by the fact that our study included a number of items with high edge density and high response time (see Figure 5).

### Within-Item Prediction of the Number of Fixations

Two regression analyses were conducted per Raven item. In the first analysis, the predictors were the edge density of the matrix cell and centrality weight of the cell (Table 2). In the second analysis, the predictors were perceived complexity of the cell and centrality weight of the cell (Table 3). It can be seen that for each of the 12 Raven items, centrality and complexity positively contributed to predicting the number of fixations on that cell. The mean predictive correlation (*r*) was 0.86 for edge density and 0.92 for perceived complexity.

**Table 2.** Results of 12 regression analyses for predicting the number of fixations on matrix cells from the edge density and centrality of the matrix cells (*n*  =  9).

Raven item	β edge density	β centrality	Predictive *r*
1 (1)	**0**.**53**	**0**.**64**	**0**.**95**
2 (4)	**0**.**39**	**0**.**77**	**0**.**97**
3 (8)	0.45	0.49	**0**.**70**
4 (11)	**0**.**95**	**0**.**64**	**0**.**91**
5 (15)	**0**.**73**	**0**.**47**	**0**.**92**
6 (18)	**0**.**55**	**1**.**07**	**0**.**95**
7 (21)	0.28	0.54	0.65
8 (23)	0.38	0.58	**0**.**71**
9 (25)	0.26	**0**.**74**	**0**.**89**
10 (30)	**0**.**70**	0.35	**0**.**90**
11 (31)	**0**.**63**	**0**.**47**	**0**.**90**
12 (35)	**0**.**65**	**0**.**47**	**0**.**92**

*Note.* Statistically significant (*p* < 0.05) regression coefficients and correlation coefficients are depicted in boldface.

**Table 3.** Results of 12 regression analyses for predicting the number of fixations on matrix cells from the perceived complexity and centrality of the matrix cells (*n*  =  9).

Raven item	β perceived complexity	β centrality	Predictive *r*
1 (1)	**0**.**54**	**0**.**58**	**0**.**95**
2 (4)	**0**.**42**	**0**.**70**	**0**.**97**
3 (8)	**0**.**67**	0.49	**0**.**86**
4 (11)	**0**.**85**	0.35	**0**.**88**
5 (15)	**0**.**78**	**0**.**37**	**0**.**94**
6 (18)	**0**.**48**	**0**.**95**	**0**.**93**
7 (21)	**0**.**76**	0.27	**0**.**91**
8 (23)	**0**.**71**	0.24	**0**.**86**
9 (25)	**0**.**61**	0.41	**0**.**95**
10 (30)	**0**.**77**	**0**.**37**	**0**.**96**
11 (31)	**0**.**69**	**0**.**45**	**0**.**94**
12 (35)	**0**.**64**	**0**.**49**	**0**.**92**

*Note.* Statistically significant (*p* < 0.05) regression coefficients and correlation coefficients are depicted in boldface.

### Repeating the Analysis While Omitting the Bottom Right Cell

The bottom right cell of each of the 12 Raven items had a centrality score of 3 and an edge density of 0. Previous research into scan paths of Raven matrices also retained the bottom right cell in the analysis (Hayes et al., 2011). However, it may be argued that the empty bottom right cell inflates the predictive correlation, because participants hardly looked at the empty cell.

In order to examine the robustness of our findings, we repeated the regression analysis by omitting the bottom right cell of each Raven item. The results of the regression analysis of the within-item analysis with the bottom right cell omitted are shown in Tables 4 and 5. The mean predictive *r* when using edge density and centrality as predictors became 0.81 (instead of 0.86 when using all nine Raven items). When using perceived complexity and centrality as predictors, the mean predictive *r* became 0.82 (instead of 0.92 when using all nine Raven items).

**Table 4.** Results of 12 regression analyses for predicting the number of fixations on matrix cells from edge density and centrality of the matrix cells, not using the bottom right matrix cell (*n*  =  8).

Raven item	β edge density	β centrality	Predictive *r*	*SD* edge density
1 (1)	0.35	**0**.**80**	**0**.**91**	0.0036
2 (4)	−0.11	**0**.**98**	**0**.**96**	0.0005
3 (8)	−0.01	0.56	0.56	0.0150
4 (11)	1.02	0.72	0.64	0.0143
5 (15)	0.59	0.77	**0**.**77**	0.0069
6 (18)	0.08	**1**.**00**	**0**.**94**	0.0056
7 (21)	−0.49	0.57	**0**.**78**	0.0140
8 (23)	−0.46	0.49	**0**.**77**	0.0089
9 (25)	0.24	**0**.**75**	**0**.**87**	0.0236
10 (30)	0.60	0.47	**0**.**85**	0.0337
11 (31)	0.00	**0**.**81**	**0**.**81**	0.0112
12 (35)	−0.32	**0**.**91**	**0**.**81**	0.0003

*Note.* Statistically significant (*p* < 0.05) regression coefficients and correlation coefficients are depicted in boldface.

**Table 5.** Results of 12 regression analyses for predicting the number of fixations on matrix cells from perceived complexity and centrality of the matrix cells, not using the bottom right matrix cell (*n*  =  8).

Raven item	β perceived complexity	β centrality	Predictive *r*	*SD* perceived complexity
1 (1)	0.37	**0**.**72**	**0**.**91**	0.660
2 (4)	0.14	**0**.**90**	**0**.**96**	0.500
3 (8)	0.31	0.65	0.64	1.011
4 (11)	0.76	0.29	0.67	1.241
5 (15)	**0**.**70**	**0**.**65**	**0**.**87**	0.740
6 (18)	0.24	**1**.**08**	**0**.**96**	0.820
7 (21)	0.43	0.39	0.71	0.440
8 (23)	0.24	0.54	0.67	1.153
9 (25)	**0**.**66**	0.36	0.96	2.049
10 (30)	**0**.**65**	**0**.**54**	**0**.**90**	1.537
11 (31)	0.02	**0**.**81**	**0**.**81**	0.409
12 (35)	–0.29	**0**.**70**	**0**.**81**	0.059

*Note.* Statistically significant (*p* < 0.05) regression coefficients and correlation coefficients are depicted in boldface.

Tables 4 and 5 show that the predictive power of centrality remained intact compared to Tables 2 and 3, with large nonzero regression coefficients. The predictive power of edge density, however, was diminished (see many negative regression coefficients in Table 4), while for perceived complexity, it remained positive (11 of the 12 regression coefficients were positive in Table 5). It should be noted, however, that all cells for some Raven items (such as Raven item 12) had identical contents, but the shapes were differently rotated. Hence, there was almost no variation in edge density or perceived complexity (see the rightmost column in Tables 4 and 5), which can explain some of the unstable regression coefficients. In summary, it can be concluded that after removing the bottom right cell of the matrices, perceived complexity and centrality, but not edge density, were still positive predictors of the number of fixations on the cells.

## Discussion

Our findings suggest the existence of visual processes that are influenced by complexity and centrality. Firstly, predictive validity of visual complexity was observed *between* the 12 Raven items, namely, Raven items that received more fixations and took a longer time to respond tended to be more complex items, as measured by both perceived complexity (obtained through crowdsourcing) and edge density (determined objectively using computer code). The association between edge density and response time held over and above the effect of item difficulty. To illustrate, participants took a long time to respond to Raven items 3, 10, and 11, and these were also the Raven items that had a high edge density. Our findings can be interpreted by stating that response times to Raven items are not just governed by cognitive load (the time required to derive the solution rules and construct the answer in mind) but also by visual load (the time required to extract the information from the stimuli, as affected by visual complexity). This finding is in line with Meo et al. (2007), who suggested that in order to solve a Raven item, the relevant elements first need to be visually encoded before applying solution steps in mind. Our findings support other research on human information processing that distinguishes between visual demands and cognitive demands (Mittelstädt et al., 2022). In car driving, for example, driving performance may be affected in different ways by cognitive load and visual load (Engström et al., 2005).

Apart from demonstrating between-item effects, the predictive validity of visual complexity was determined *within* the individual Raven items; specifically, the number of fixations that fell on a matrix cell was related to the perceived complexity of the cell, with the most extreme case being an empty cell receiving 35 times fewer fixations than a visually complex cell. Within Raven items, perceived complexity turned out to be a better predictor than edge density, presumably because cells in Raven items often had similar edge density.

Apart from complexity, participants’ visual attention was governed by centrality, with the middle cell of the matrix receiving about 60% more fixations than the edge cells. In summary, our findings suggest that human responses to Raven matrices are not just governed by cognitive demands but also by visual demands, which is supportive of previous research into Raven matrices by Meo et al. (2007) and Hua and Kunda (2020). Thus, although Raven matrices are often portrayed as a test of pure cognitive reasoning, responses to Raven matrices are not purely cognitive but are strongly governed by visual features. Some Raven items seem to be easier than others because they can be solved using Gestalt perception, i.e., nearly automatically, while more difficult Raven items require a careful assessment of the cell content.

The observation that participants approach Raven problems by making many fixations in a stepwise manner is not novel. According to an eye-tracking study by Carpenter et al. (1990), test-takers derive solution rules from small steps consisting of pairwise comparisons between adjacent cells. More capable test-takers break down the Raven item into smaller subproblems, which they then process incrementally through pairwise comparison. The novelty of the current study lies in its discovery that the extent of incremental search (i.e., the number of fixations on cells) is largely influenced by quantifiable visual features of the cells, as determined either by a computer algorithm (edge density) or human ratings of complexity.

A limitation of our research is that the visual complexity of Raven items was quantified by letting human raters compare two matrix cells in isolation, without considering visual patterns in the Raven item as a whole. Future research could measure the complexity of a group of cells or of entire Raven items. Another limitation is that our findings were obtained among a sample of capable students and under conditions of considerable time pressure. Participants provided an average of 7.30 correct responses, which indicates high ability. In comparison, in the study by Arthur et al. (1999), university participants (*n*  =  1506) answered 7.73 Raven items correctly while using more time (15 min compared to 7 min in our case). In other studies that used the same 12-item version with university samples and a 15-min time limit, the mean number of correct responses ranged between 6.62 (Arthur & Day, 1994; Van der Leer et al., 2015) and 8.28 (Von Bastian et al., 2016). These observations are consistent with Wai et al. (2009), who noted that engineering MSc students excel in spatial abilities, including tests of abstract reasoning. Future studies should investigate the effects of visual complexity on a broader range of cognitive tests and diverse populations, including individuals with various abilities, backgrounds, and age groups, to enhance our understanding of its impact on test performance. This research can be further supported by creating alternative methods for measuring the complexity of test items and exploring different techniques to assess cognitive processing while solving Raven matrices, such as using brain imaging (Yuan et al., 2012).

Another limitation of this study is that it was conducted with a time limit of 7 min. It is common to perform Raven matrices with a time limit, even with a stricter time limit than our current study (e.g., 18 items with a 10-min time limit: Ellis et al., 2021; Robison & Campbell, 2023; Tsukahara & Engle, 2021). Based on an overview of studies in the Supplementary Material, there seem to be some indications that if no time limit is used (as the Raven matrices are supposed to be administered), response time is more determined by item difficulty. For example, Liu et al. (2022) found that the mean response time of the 36 items strongly correlated (*r*  =  0.93) with item difficulty, defined as the percentage of responses that were correct. This suggests that when participants have and take ample time, visual complexity becomes a less determining factor for response time, while the difficulty of the item becomes more decisive.

Considering the implications of our study, the current findings emphasize the need for test designers to consider visual complexity and centrality when designing cognitive tests. We showed that test items that are visually more complex or that require a large amount of visual scanning are expected to result in longer response times, potentially affecting test scores. To improve the validity of cognitive tests, test designers are advised to design items that maintain a controlled level of visual complexity and minimize the necessity for extensive visual scanning. This approach may contribute to a more precise evaluation of cognitive capabilities.

In addition, the present study holds implications for researchers in the field of psychometrics as well as those with an interest in comprehending intelligence. Intelligence is typically defined as the capacity to undertake complex cognitive processing, which encompasses abstract reasoning and logical deduction (Gottfredson, 1997). The Raven Progressive Matrices are specifically designed to evaluate such processing. However, our research has revealed that elementary visual complexity also contributes to performance on this test. On the one hand, it can be argued that the incorporation of rudimentary visual elements is essential in intelligence evaluations. Luria's (1966) neuropsychological theory of intelligence underscores the role of sensory processes in cognitive functions. In accordance with his research, sensory processes are the basis for higher cognitive operations, and intelligence is not solely a consequence of abstract reasoning. On the other hand, low-level visual tasks like visual search and visual comparison have only a weak correlation with IQ scores (Marshalek et al., 1983). Consequently, the integration of visually intricate Raven items, necessitating extensive visual scanning, may diminish the efficiency of the test. From a broader perspective, our study highlights the importance of considering not only the conceptual complexity of a task but also its visual complexity, as both factors could influence the efficiency with which individuals process and respond to information.

## Supplemental Material

sj-docx-1-pec-10.1177_03010066231178149 - Supplemental material for Responses to Raven matrices: Governed by visual complexity and centrality Click here for additional data file. Supplemental material, sj-docx-1-pec-10.1177_03010066231178149 for Responses to Raven matrices: Governed by visual complexity and centrality by Joost C. F. de Winter, Dimitra Dodou, and Yke Bauke Eisma in Perception
