# Supplementary material for: Responses to Raven matrices: Governed by visual complexity and centrality
Source: Perception. 2023 Jun 2;52(9):645–61. doi: 10.1177/03010066231178149 (PMC10469510; doi:10.1177/03010066231178149)
Supplement: sj-docx-1-pec-10.1177_03010066231178149 - Supplemental material for Responses to Raven matrices: Governed by visual complexity and centrality [file sj-docx-1-pec-10.1177_03010066231178149.docx]

**Supplementary Material**

De Winter, J. C. F., Dodou, D., & Eisma, Y. B. (2023). Responses to Raven matrices: Governed by visual complexity and centrality? Perception. <https://doi.org/10.1177/03010066231178149>

**Measuring Perceived Complexity Through Crowdsourcing**

A total of 775 participants were recruited through the Prolific crowdsourcing platform. The survey was titled “*Assessing the complexity of shapes*”. A brief description of the study was provided, followed by a question asking for informed consent. Participants who did not give consent were directed to a page thanking them for considering the study. Participants were allowed to participate only once. The research was approved by the Human Research Ethics Committee of the Delft University of Technology.

When a study is published in Prolific, an email is sent to a number of randomly selected eligible participants based on their demographics. Participants can also see studies for which they are eligible on their Prolific dashboard or corresponding app.

Participants answered a number of demographic questions, after which they were provided with a hyperlink to Qualtrics to perform the experiment. First, the following instruction was presented:

“*The aim of the study is to investigate how humans perceive shape complexity. You will view 150 pairs of shapes, side-by-side. For each pair, you will be asked which of the two shapes is, according to your view, more complex. There is no right or wrong answer. Please take your time and judge the shapes carefully. Press ‘Start’ to proceed with the first pair of shapes.*”

Next, the participants were presented with a pair of shapes (matrix cells in the same grayscale as used in the eye-tracking experiment). Above the pair, the count of the comparison (e.g., “*1 of 150*”) was presented, as well as the question: “*Which of the two shapes is more complex?*”. Participants could choose one of the two shapes by clicking on it with their mouse; a light blue frame appeared briefly around the chosen shape, and the next pair automatically appeared.

After completing all 150 comparisons, participants were asked to respond to the following open question: “*Could you please describe what strategies you used when assessing complexity?*” (these data were not used in the present study). On the final page, participants were thanked for their participation and redirected back to Prolific to register their submission and receive their reimbursement. Participants received a reimbursement of 1.8 GBP (~ 2 Euro) for an estimated 12-minute duration of the experiment. This corresponds to an hourly rate of 9 GBP (~10.2 Euro), which Prolific characterizes as ‘good’.

Of the 775 participants, 29 did not give consent, and 147 did not complete all 150 comparisons and were excluded, leaving 599 participants for the analysis. Accordingly, a total of 89,850 (599 participants x 150 responses) responses to the paired comparisons were obtained. The mean completion time was 640.7 s (*SD* = 329.5 s, median = 566 s). The participants resided in 29 countries. The five most represented countries were Portugal (*n* = 122), Poland (*n* = 95), South Africa (*n* = 80), Mexico (*n* = 71), and Italy (*n* = 54). The sample consisted of 312 (52.1%) male participants, 278 (46.4%) female participants, and 9 (1.5%) participants who answered ‘I prefer not to respond’. The mean age was 28.5 years (*SD* = 9.7 years, median = 25 years, range = 19–69 years).

**Statistics per Item**

The number of correct responses, number of incorrect responses, percentage of correct responses, number of no responses, and mean and *SD* response times per item are provided in Table S1.

Table S1

*Descriptive statistics of the 12 Raven items used in the experiment*

| **Raven item** | **Correct**  **response provided**  **(*n*)** | **Incorrect**  **response provided**  **(*n*)** | **Correct**  **response provided**  **(%)** | **No response provided**  **(*n*)** | **Mean**  **response**  **time (s)** | ***SD***  **Response**  **time (s)** |
| --- | --- | --- | --- | --- | --- | --- |
| 1 (1) | 141 | 18 | 89 | 0 | 21.1 | 12.9 |
| 2 (4) | 131 | 28 | 82 | 0 | 22.0 | 12.9 |
| 3 (8) | 129 | 30 | 81 | 0 | 33.3 | 19.8 |
| 4 (11) | 136 | 23 | 86 | 0 | 23.2 | 13.1 |
| 5 (15) | 113 | 46 | 71 | 0 | 28.6 | 12.4 |
| 6 (18) | 120 | 39 | 75 | 0 | 26.8 | 14.3 |
| 7 (21) | 84 | 74 | 53 | 1 | 35.4 | 15.9 |
| 8 (23) | 92 | 61 | 60 | 6 | 37.1 | 19.4 |
| 9 (25) | 85 | 63 | 57 | 11 | 35.3 | 17.1 |
| 10 (30) | 53 | 76 | 41 | 30 | 53.1 | 25.3 |
| 11 (31) | 42 | 57 | 42 | 60 | 54.3 | 25.7 |
| 12 (35) | 34 | 39 | 47 | 86 | 35.5 | 15.3 |
|  |  |  |  |  |  |  |

**Correlation Matrix**

Table S2 shows the correlation coefficients between Raven performance and response times. According to the speed-accuracy trade-off, participants can increase the percentage of correct responses by taking more time per Raven item (e.g., Goldhammer et al., 2015). Consistent with this strategy, a moderate positive correlation (*r* = 0.44, *p* < 0.001) was found between the mean response time and the percentage of correct responses.

Table S2 also shows that participants who spent a greater proportion of time looking at the matrix (and less time looking at the response alternatives) provided more correct responses. This association existed regardless of whether the number of correct responses was considered or the proportion of correct responses.

Table S2

*Descriptive statistics and intercorrelations between measures (n = 159)*

|  | ***M*** | ***SD*** | **1** | **2** | **3** | **4** |
| --- | --- | --- | --- | --- | --- | --- |
| 1. Number of correct responses (*n*) | 7.30 | 1.93 |  |  |  |  |
| 2. Proportion of responses that are correct (%) | 0.69 | 0.20 | **0.86** |  |  |  |
| 3. Response time (s) | 33.53 | 8.10 | 0.04 | **0.44** |  |  |
| 4. Gaze time on matrix area (% of time) | 74.32 | 5.59 | **0.17** | **0.29** | **0.31** |  |
| 5. Gaze time on response alternatives (% of time) | 23.34 | 4.11 | **-0.24** | **-0.33** | **-0.27** | **-0.64** |

*Note.* Statistically significant correlations (*p* < 0.05) are marked in boldface.

**Edge Density and Response Time Extracted from Other Studies**

Based on scans of the RAPM booklet (Raven, 1976), the edge density per Raven item was computed. The edge density values are shown in Figure S1 and were highly similar to the edge densities of the redrawn Raven matrices as used in the paper (see Figure S2 for a scatter plot).

Next, mean response times were extracted from published papers that used a computerized version of the RAPM. The results in Figure S3 illustrate that later Raven items generally took a longer time to respond. The item number (1 to 36) was also found to correlate positively with the item edge density (*r* = 0.32, *p* = 0.055, *n* = 36). Visual complexity (edge density) is predictive of mean item response time, even when item difficulty (item number) is corrected for (Table S3).

*Figure S1.* Edge density for all 36 items of the Raven Advanced Progressive Matrices.

*Figure S2.* Edge density for the Raven item means of the redrawn cells used in the analysis versus edge density from screenshots of the scans from the RAPM booklet (Raven, 1976). The depicted correlation (*r*) is 0.999.

*Figure S3.* Mean response times for items of Raven’s Advanced Progressive Matrices for the current study and for lab experiments from the literature. The magenta dotted line represents a linear fit of item number and grand mean response time. It can be seen that more difficult items (i.e., higher item number) generally took longer for participants. For Ellis et al. (2021), Frischkorn and Von Bastian (2021), Loesche et al. (2015), Poulton et al. (2022), Robison and Campbell (2023), and Tsukahara and Engle (2021), very fast response times, specifically responses faster than 4 seconds, are excluded from the calculation of the mean response time. The reason is that in these studies, there are indications that a portion of participants, especially towards the end of the test, quickly scrolled through the items.

Table S3. *Correlation coefficients between Raven item number and mean response time, correlation coefficient between edge density and median response time, and partial correlation between edge density and mean response time while controlling for item number.*

|  | **Number of items** | **Time**  **constraint** | ***r***  **item number** | ***r***  **edge density** | **Partial *r***  **edge density** |
| --- | --- | --- | --- | --- | --- |
| 1. Current study | 12 | 7 min | **0.79** | **0.82** | **0.80** |
| 2. Adam and Vogel (2018) | 36 | 10 min for 18 items | **0.70** | **0.44** | 0.32 |
| 3. Ellis et al. (2021) | 18 | 10 min | **0.70** | **0.53** | 0.47 |
| 4. Frischkorn and Von Bastian (2021) | 12* | 15 min | **0.87** | **0.73** | **0.70** |
| 5. Sense et al. (2019) | 36 | 10 min for 18 items | **0.68** | **0.51** | **0.42** |
| 6. Goldhammer et al. (2015) | 36 | unlimited | **0.87** | **0.33** | 0.11 |
| 7. Gonthier & Roulin (2020) | 36 | unlimited | **0.82** | **0.46** | **0.37** |
| 8. Liu et al. (2022) | 36 | unlimited | **0.87** | 0.27 | -0.02 |
| 9. Loesche et al. (2015), Study 1–4 | 26 | unlimited | **0.51** | **0.48** | 0.37 |
| 10. Poulton et al. (2022), Study 1 | 12* | unlimited | **0.76** | **0.63** | 0.45 |
| 11. Poulton et al. (2022), Study 2 | 12* | 60 s per item | **0.80** | **0.68** | 0.53 |
| 12. Rivollier et al. (2021), Study 1 | 12* | unlimited | **0.64** | **0.66** | 0.51 |
| 13. Robison and Campbell (2023) | 18 | 10 min | **0.78** | 0.46 | 0.38 |
| 14. Tsukahara and Engle (2021), Study 1 | 18 | 10 min | **0.86** | 0.43 | 0.37 |
| 15. Tsukahara and Engle (2023) | 18 | 10 min | **0.82** | 0.43 | 0.34 |
| 16. Vigneau et al. (2006) | 14 | unlimited | **0.82** | **0.56** | **0.58** |

*Note.* Statistically significant correlations (*p* < 0.05) are marked in boldface. For Frischkorn and Von Bastian (2021), only the ‘pre-intervention’ data were used. For Rivollier et al. (2021), participants performed a ‘switch’ condition, which entailed that either only the matrix or only the response options were visible.

* Same items as the current study.

**Number of Fixations per Cell**

Table S4 shows the mean number of fixations per matrix cell. The middle cell received the most attention, while the edge cells (top left, top right, bottom left) received much less attention. It can also be seen that there were substantial differences between Raven items. The matrices of Raven items 10 and 11 received the most fixations.

Table S4. *Participants’ mean number of fixations per matrix cell and total number of fixations per Raven item. The bottom row lists the mean of the 12 Raven items.*

*Note.* The cell is filled linearly based on the depicted value.

Our study examined participants’ attention distribution across the cells of the problem matrix. An assessment of fixations on the response alternatives (Table S5) showed that participants focused mainly on the response they provided (which was usually the correct response for the earlier items). An interesting observation was that for some items, such as item 5, participants focused relatively often on the response alternatives and relatively little on the matrix. A possible explanation is that the matrix featured Gestalt-like elements (e.g., two semicircles adding up to a full circle), but it was hard to eliminate response alternatives (e.g., response alternatives of this item looked similar and therefore required a detailed examination).

Table S5. *Participants’ mean number of fixations per response alternative. Also shown is the percentage of fixations on the response alternatives that fell on the correct response.*

*Note.* The cell is filled linearly based on the depicted value.
